# New method for determining breast cancer recurrence-free survival using routinely collected real-world health data

**DOI:** 10.1186/s12885-022-09333-6

**Published:** 2022-03-16

**Authors:** Hyunmin Jung, Mingshan Lu, May Lynn Quan, Winson Y. Cheung, Shiying Kong, Sasha Lupichuk, Yuanchao Feng, Yuan Xu

**Affiliations:** 1 Department of Economics, Faculty of Arts, University of Calgary, 2500 University Dr. NW, Calgary, AB T2N 1N4 Canada; 2 Department of Community Health Sciences, Cumming School of Medicine, University of Calgary, 3D10 3280 Hospital Drive NW, Calgary, AB T2N 4Z6 Canada; 3 Department of Surgery, Cumming School of Medicine, University of Calgary, North Tower, Foothills Medical Centre, 1403 29 St NW, Calgary, AB T2N 2T9 Canada; 4 Department of Oncology, Cumming School of Medicine, University of Calgary, Tom Baker Cancer Centre, 1331 29th St NW, Calgary, AB T2N 4N2 Canada; 5 Centre for Health Informatics, Cumming School of Medicine, University of Calgary, Teaching Research and Wellness (TRW), 5E04 Hospital Dr NW, Calgary, AB T2N 4Z6 Canada

**Keywords:** Breast cancer, Survival analysis, Timing of recurrence, Identification algorithm, Real-world data

## Abstract

**Background:**

In cancer survival analyses using population-based data, researchers face the challenge of ascertaining the timing of recurrence. We previously developed algorithms to identify recurrence of breast cancer. This is a follow-up study to detect the timing of recurrence.

**Methods:**

Health events that signified recurrence and timing were obtained from routinely collected administrative data. The timing of recurrence was estimated by finding the timing of key indicator events using three different algorithms, respectively. For validation, we compared algorithm-estimated timing of recurrence with that obtained from chart-reviewed data. We further compared the results of cox regressions models (modeling recurrence-free survival) based on the algorithms versus chart review.

**Results:**

In total, 598 breast cancer patients were included. 121 (20.2%) had recurrence after a median follow-up of 4 years. Based on the high accuracy algorithm for identifying the presence of recurrence (with 94.2% sensitivity and 79.2% positive predictive value), the majority (64.5%) of the algorithm-estimated recurrence dates fell within 3 months of the corresponding chart review determined recurrence dates. The algorithm estimated and chart-reviewed data generated Kaplan–Meier (K-M) curves and Cox regression results for recurrence-free survival (hazard ratios and *P*-values) were very similar.

**Conclusion:**

The proposed algorithms for identifying the timing of breast cancer recurrence achieved similar results to the chart review data and were potentially useful in survival analysis.

**Supplementary Information:**

The online version contains supplementary material available at 10.1186/s12885-022-09333-6.

## Background

Breast cancer is the second most common cancer among Canadian women [1] and 5-year breast cancer-specific survival is about 88% overall and higher for those with stage 0 and 1 disease [2]. While breast cancer incidence and mortality are carefully tracked by provincial registries, cases of recurrent disease (local–regional and/or distant metastases) are not systematically captured. Recurrence is a much more common event compared with breast cancer mortality and hence, recurrence-free survival (RFS) is an important outcome to study. RFS analyses based on large scale real-world data enable efficient evaluation of the impact of new surgical, radiation, and systemic treatments; identification of regional gaps in care; benchmarking progress across jurisdictions; and better planning of resources and clinical trials for those with relapsed disease. However, studying population-based outcomes for early breast cancer, and breast cancer that has relapsed, is challenging as it requires chart review which is time consuming and costly [3].

Recurrence of breast cancer is an event which usually requires intensive health care resources such as advanced diagnostic imaging, biopsy, re-operation, and additional radiation and systemic therapies. This is often characterized by a sudden increase in frequency of medical encounters such as surgical oncologist or cancer center visits, or a new round of treatments. In a universal health system, the routinely collected population-based real-world health data, which capture the entire disease trajectory of a patient, provides a potential source to determine these changes in pattern of care. Further, these identified changes in care can be used to develop methods to determine whether and when the cancer has recurred. Thus, this offers a possibility to develop an algorithm that relies on the pattern of cancer care using population-based health data to identify breast cancer recurrence and its timing.

Although several studies (mainly in the United States) have used procedure and diagnostic codes to detect and identify cancer recurrence status [4–12], most of these algorithms were not developed to identify timing of cancer recurrence. A few studies [5, 10–12] addressed the challenge of identifying timing of breast cancer recurrence, however, due to differences in study cohorts, health systems (universal vs. mixed market system), and data coding standards (e.g. the Current Procedural Terminology is only used in the United States data) between their data and ours, these previously developed algorithms are not applicable to be implemented in our data. Thus, we developed a set of algorithms to identify the status of recurrence of breast cancer in our previous study [13]. However, with the lack of timing of recurrence, conducting the time-to-event RFS analysis is impossible, therefore, developing methods to identify the timing of recurrence is needed. This study aimed to develop and validate the methods for identifying timing of recurrence and then the length of RFS using real-world data from a health system with universal insurance coverage.

## Methods

### Data sources and study cohort

We used the data from two previously chart-reviewed cohorts with known high breast cancer recurrence rates [14, 15]. The young women cohort consisted of patients who were under the age of 41 and were diagnosed with breast cancer between 2007 and 2010. The neoadjuvant chemotherapy cohort consisted of neoadjuvant chemotherapy patients who were diagnosed with breast cancer between 2007 and 2014. Both cohorts were limited to those who were diagnosed in Alberta, Canada during the specified time periods. Patients who did not have an Alberta health care number, moved out of province within 1 year of surgery, had more than one type of tumor (i.e. second primary cancer), or had stage IV breast cancer were excluded from the study.

Our study cohorts were obtained from the Alberta Cancer Registry (ACR). The ACR is a population-based registry operated by the Alberta Health Service (AHS)—Cancer Care that records and maintains information of all cancer patients in the province such as patient name, sex, age, type of tumor, tumor characteristics (e.g., tumor stage, histology and biomarker subtypes), date of cancer diagnosis/primary treatment. Follow-up information was derived from multiple other provincial datasets. These datasets included the provincial wide cancer center electronic medical record (EMR), physician claims and vital statistics from Cancer Care Alberta and the Department of Analytics of AHS. The cancer center EMR includes the type and date of cancer center visits (e.g., chemotherapy, radiation therapy, oncologist consultation). The physician claims data records the type and dates of any procedures (e.g., diagnostic imaging, biopsy, and surgery) delivered to the patients. The vital statistics data records death and cause of death (e.g., breast cancer-related death). All available real-world health care data was linked to depict the entire care trajectory of each patient. We used the linked data to acquire patient’s treatment information such as the dates of the episode of chemotherapy and radiation therapy, the dates of outpatient/inpatient visits and the cause of death.

The patient’s recurrence status (i.e., no recurrence, local or distant recurrence) and recurrence date were ascertained by chart review and these served as gold standards to validate our developed algorithms. Chart review and abstraction were performed by MLQ and a general surgery fellow. MLQ is an experienced general surgeon and researcher, who trained the surgery fellow to conduct the chart review. Then the surgery fellow worked independently. Cases where data were unclear or ambiguous at the time of primary data entry were reviewed by both MLQ and the fellow with outcomes agreed upon by consensus. The chart review covered from patient’s initial curative-intent treatment to first recurrence, or death, or the last follow-up date (i.e., September 1, 2017). The individual patient’s follow-up time frame considered in the algorithm was the same as the one in the chart review.

### Definition of breast cancer RFS

For chart review, we defined the recurrence as the development of in-situ or invasive tumors in the breast, lymph nodes or at a distant site occurring after 180 days or more from the definitive surgical date. Patients who developed second primary cancer (e.g., contralateral breast cancer) after the primary treatment determined by chart review were excluded to increase the applicability of our study.

The study outcome was RFS (i.e., recurrence and timing of recurrence) of breast cancer. Whether the recurrence status was estimated by the algorithm or derived from the chart review, patients who were regarded as having recurrence were coded as “Yes”. All the other patients including the dead patients who did not experience recurrence were coded as value “No”. Using the date (either chart review determined or algorithms estimated) of the recurrence and the date of surgery, the RFS was defined as the period between the surgery date and the recurrence date.

### Indicators of recurrence

We assumed that a second cluster of clinical visits and post-primary treatments occurring at least 6 months after the definitive surgical date were potential indicators of recurrence. Since primary treatment completion generally took longer than 6 months, we also tested the algorithm using other time intervals including 12 months and 18 months after the definitive surgical date. The clinical visits and post-primary treatments considered included: diagnostic imaging tests (e.g., mammography), biopsy, surgery (e.g., mastectomy or BCS), radiation, chemotherapy, and hormonal therapy. We observed patients’ trajectories from their primary definitive surgical date and created separate indicator variables coded as ‘yes’ or ‘no’ for whether the number of patient’s clinical visits exceeded a prespecified value (e.g. 3 times), whether the patient went through another set of diagnostic procedures (imaging, biopsy), whether the patient went through another set of local or regional treatments (breast surgery, radiation), and whether the patient underwent more than two chemotherapy cycles. We also included living or deceased status of a patient, cause of death, and stage of tumor as indicators of recurrence in the developed algorithms. All the codes and data sources used for defining the study variables were presented in the Additional file Table 1.

### Identifying the date of recurrence and length of recurrence-free survival

Similar with other studies [4], our previously developed recurrence identification algorithms were classification and regression tree (CART)-based decision trees [16, 17] which incorporated the indicators described above. Each indicator was a node in the tree and split the cohort into recurrent (i.e. meet the indicator) and non-recurrent cases (i.e. failed to meet the indicator). For instance, if the patient had second chemotherapy 1 year after the primary surgery (indicator), then the patient was classified as breast cancer recurrence case, otherwise not. Patients who were classified as non-recurrent cases would then be further classified using another indicator (or node). Similarly, this node split the cohort into a recurrent case or a non-recurrent case. Carrying on in the same fashion, the patient would be triaged into a non-recurrent or current case until the bottom of the decision tree. The CART decision trees were optimized by choosing a splitting node (indicator) that minimized the Gini index that is the most commonly used index for classification problems.

We also validated the decision trees accepting the chart review data as the reference. The detailed description of the development of the decision trees can be found in our previously published paper [13]. Considering different utilizations of the recurrence algorithms, we developed a set of algorithms (i.e., decision trees) including the high sensitivity algorithm (i.e., identifying as many true recurrent cases as possible), high positive predictive value (PPV) algorithm (i.e., ensuring as many identified recurrent cases are true cases as possible), and high overall accuracy algorithm (i.e., balancing sensitivity and positive predictive value). The high sensitivity algorithm reached 94.2% sensitivity, 93.7% specificity, 79.2% PPV, and 98.5% negative predictive value (NPV). The high PPV algorithm achieved 75.2% sensitivity, 98.3% specificity, 91.9% PPV, and 94% NPV. The high accuracy algorithm for identifying recurrence reached 85.1% sensitivity, 97.3% specificity, 88.8% PPV, 96.3% NPV, and 94.8% overall accuracy. The details of the decision trees including the construction of each indicator (code and definition) were presented in our published paper [13] and also in Additional files 2, 3, and 4.

Based on each of the three algorithms (high sensitivity, high Positive Predictive Value (PPV) and high overall accuracy), we estimated the timing of breast cancer recurrence. Our algorithms estimated a patient as a recurrent case when the patient met one of the indicators (i.e. nodes) in the decision tree. Some of the indicators in the decision tree have a specific date (e.g. the second surgery), thus, its date was used to estimate the date of the recurrence for the patients who met the indicator. For the indicators that did have a specific date but contain a time frame (e.g. a new cluster of cancer center visits), we estimated the date of recurrence as the middle point of the time frame if the patients met the indicator. Using the algorithm-estimated recurrence date and the primary surgery date, we calculated the length of RFS. For the five patients (0.8%) who had no surgery, the diagnosis date was used for RFS calculation. Those who were defined as non-recurrent cases by the algorithm were considered censored at their last known date in the survival analyses.

After we applied each of the three algorithms, we obtained three groups of recurrent patients (with corresponding non-recurrent patients). This also generated three sets of data (i.e. patient/tumor characteristics, treatments, and outcomes) based on each recurrent groups determined by one specific algorithm. For the purpose of validation, we compared the algorithm estimated length of RFS with that of chart review at the individual patient level. In addition, to conceptualize how the algorithms perform in real applications, we also investigated the agreement between each algorithm estimated group with the chart review group (reference) in terms of the patient characteristics and the results of survival analyses.

### Statistical analysis

The descriptive analysis was performed to compare the characteristics (e.g. age, tumor characteristics, and treatment received) of the group of chart-review determined recurrent patients with that of the non-recurrent patients. These comparisons were also conducted between the chart review determined and the algorithm estimated recurrence cohort (Table 1). Using the T-test for the continuous variable (or Wilcoxon test when data was not normally distributed), and the Chi-squared or Fisher exact test for the categorical variables, we tested the differences between recurrent and non-recurrent patients. The similar comparison was conducted between the algorithm-estimated recurrent patients and non-recurrent patients (Table 1).

**Table 1:** The comparison of characteristics between chart review and algorithm determined cohort

Variable		Chart-review determined			Algorithm estimated recurrence					
		**Entire cohort (*N* = 598) N(%)**	**Recurrence (*N* = 121) N(%)**	***P*-value**	**High-accuracy (*N* = 116) N(%)**	***P*-value^**	**High-PPV (*N* = 99) N(%)**	***P*-value^**	**High-sensitivity (*N* = 144) N(%)**	***P*-value^**
**Age (year)**	Median (IQR)	40 (36–53)	40 (36–50)	0.545	40 (37–53)	0.436	40 (37–50)	0.883	40 (36–53)	0.405
Stage*	0-I	95 (15.9)	9 (7.4)	< 0.0001	5 (4.3)	0.423	6 (6.1)	0.598	10 (6.9)	0.921
	II	311 (52)	41 (33.9)		39 (33.6)		30 (30.3)		51 (35.4)	
	III	192 (32.1)	71 (58.7)		72 (62.1)		63 (63.6)		83 (57.6)	
**PR**	Negative	223 (37.3)	51 (42.1)	0.216	54 (46.6)	0.332	41 (41.4)	0.89	63 (43.8)	0.688
	Positive	375 (62.7)	70 (57.9)		62 (53.4)		58 (58.6)		81 (56.3)	
**ER**	Negative	159 (26.6)	37 (30.6)	0.266	40 (34.5)	0.364	31 (31.3)	0.878	47 (32.6)	0.596
	Positive	439 (73.4)	84 (69.4)		76 (65.5)		68 (68.7)		97 (67.4)	
HER2*	Negative	437 (73.1)	98 (81)	0.028	93 (80.2)	0.82	79 (79.8)	0.761	112 (77.8)	0.324
	Positive	161 (26.9)	23 (19)		23 (19.8)		20 (20.2)		32 (22.2)	
**Tumor histology**	Ductal	538 (90)	106 (87.6)	0.349	98 (84.5)	0.595	83 (83.8)	0.478	125 (86.8)	0.949
	Lobular	27 (4.5)	5 (4.1)		6 (5.2)		6 (6.1)		6 (4.2)	
	Others	33 (5.5)	10 (8.3)		12 (10.3)		10 (10.1)		13 (9.1)	
**Tumor grade**	1	45 (7.5)	5 (4.1)	0.284	6 (4.2)	0.024	4 (4.0)	0.04	4 (3.5)	0.038
	2	211 (35.3)	44 (36.3)		46 (31.9)		30 (30.3)		35 (30.2)	
	3	342 (57.2)	72 (59.5)		92 (63.9)		65 (65.7)		77 (66.4)	
Surgery*	No surgery	5 (0.8)	1 (0.8)	0.002	1 (0.9)	0.370	1 (1)	0.052	5 (3.5)	0.135
	BCS	159 (26.6)	18 (14.9)		13 (11.2)		7 (7.1)		23 (16)	
	Mastectomy	434 (72.6)	102 (84.3)		102 (87.9)		91 (91.9)		116 (80.6)	
**Chemotherapy**	No	51 (8.5)	10 (8.3)	0.614	7 (6)	0.377	7 (7.1)	0.658	15 (10.4)	0.357
	Yes	547 (91.5)	111 (91.7)		109 (94)		92 (92.9)		129 (89.6)	
**Hormone therapy**	No	192 (32.1)	43 (35.5)	0.396	36 (31)	0.397	32 (32.3)	0.63	49 (34)	0.762
	Yes	214 (35.8)	37 (30.6)		34 (29.3)		29 (29.3)		42 (29.2)	
	Unknown	192 (32.1)	41 (33.9)		46 (39.7)		38 (38.4)		53 (36.8)	
**Radiotherapy**	No	209 (34.9)	40 (33.1)	0.587	36 (31)	0.658	31 (31.3)	0.718	48 (33.3)	0.573
	Yes	194 (32.4)	44 (36.4)		40 (34.5)		34 (34.3)		47 (32.6)	
	Unknown	195 (32.6)	37 (30.6)		40 (34.5)		34 (34.3)		49 (34)	

* indicates the statistically significant difference (*P* < 0.05) between the recurrent and non-recurrent patients by univariate analysis based on chart review data

^ The *p*-values is for the statistical comparison between algorithm-estimated recurrence cohort and the chart review determined recurrence cohort

To assess the validity of the estimated date of recurrence, we compared the algorithm-estimated recurrence date with the real recurrence date (i.e. the chart review defined) for each patient. Similar with previous study [10–12], taking the difference between the estimated and real recurrence date, we then classified the absolute difference (in months) into the corresponding interval (e.g. 0–1, 1–2, 2–3, 3–6, or > 6 months) and constructed a frequency table to examine the agreement between the estimated and real recurrence date. In addition, using the recurrence date and primary surgery date we calculated the length or RFS then compared the estimated with the real length of RFS using Wilcoxon test given the skewed distribution of the length of RFS.

To further assess the agreement of the survival analysis between the algorithm-estimated and the real recurrence and timing, we created Kaplan-Meyer (K-M) curves of RFS using the algorithm-estimated and the chart review data accordingly. In addition, separate Cox regressions (modeling recurrence-free survival) were performed to compare the hazard ratio and the p-value of each independent variable between the estimated data and chart review data.

All analyses were made using SAS 9.4 (SAS Institute Inc., Cary, NC). The statistical significance was set at 5% level (two-sided).

## Results

A total of 598 patients with stage 0 to III breast cancer were included and analyzed in our study. The entire cohort was composed of 282 (47.2%) young patients (less than 41 years) and 316 (52.8%) neoadjuvant chemotherapy patients. Among these patients, breast cancer recurred for 121 (20.2%) patients during a median follow-up of 4 (Interquartile Ratio (IQR) 3–5) years. The univariate analysis showed statistically significant differences between recurrent and non-recurrent patients in stage of tumor, tumor grade, human epidermal growth factor receptor-2 (HER2) status, surgery type (e.g. breast-conserving surgery (BCS), mastectomy), cancer specific death and overall death. On the contrary, tumor characteristics such as histology subtype and hormone receptor (HR) status, and patient/treatment characteristics such as age, adjuvant therapies, and follow-up length were not significantly different between recurrent and non-recurrent patients. All the three algorithm-estimated recurrence cohorts were similar to the chart review determined recurrence cohort in terms of the patient, tumor and treatment characteristics, except for the tumor grade (Table 1).

As shown in Table 2, the majority of the algorithm-estimated recurrence dates fell within 3 months of the corresponding chart review determined recurrence dates. Specifically, the proportions of patients with the difference between the algorithm-estimated and chart review date of recurrence among the 121 recurrent patients falling within 3 months were 71.1% (86) for high sensitivity algorithm, 60.3% (73) for high PPV algorithm, and 64.5% (78) for high accuracy algorithm, respectively. The Wilcoxon test showed that there was no significant difference between the chart review and algorithm-estimated length of RFS, the p-value was 0.205 for high sensitivity algorithms (vs. chart review), 0.608 for high PPV algorithms, 0.429 for high-accuracy algorithm, respectively. In addition, when only considering the 121 recurrent (determined by chart review) patients, there was also no significant difference between the algorithm-estimated and chart review determined length of RFS.

**Table 2:** The difference between algorithm-estimated and real chart review determined date of recurrence (*N* = 121)*

Absolute difference from chart review (month)	High-sensitivity algorithm		High-PPV algorithm		High-accuracy algorithm	
	**N (%)**	**Cumulative N (%)**	**N (%)**	**Cumulative N (%)**	**N (%)**	**Cumulative N (%)**
** < = 1**	51 (42.1)	51 (42.1)	41 (33.9)	41 (33.9)	47 (38.8)	47 (38.8)
** > 1, < = 2**	24 (19.8)	75 (62.0)	22 (18.2)	63 (52.0)	22 (18.2)	69 (57.0)
** > 2, < = 3**	11 (9.1)	86 (71.1)	10 (8.3)	73 (60.3)	9 (7.4)	78 (64.5)
** > 3, < = 6**	12 (9.9)	98 (81.0)	16 (13.2)	89 (73.5)	14 (11.5)	92 (76.0)
** > 6**	23 (19.0)	121 (100.0)	32 (26.5)	121 (100.0)	29 (24.0)	121 (100.0)

*RFS* recurrence-free survival, *PPV* positive predictive value

* This was determined based on chart review

**Fig. 1:**
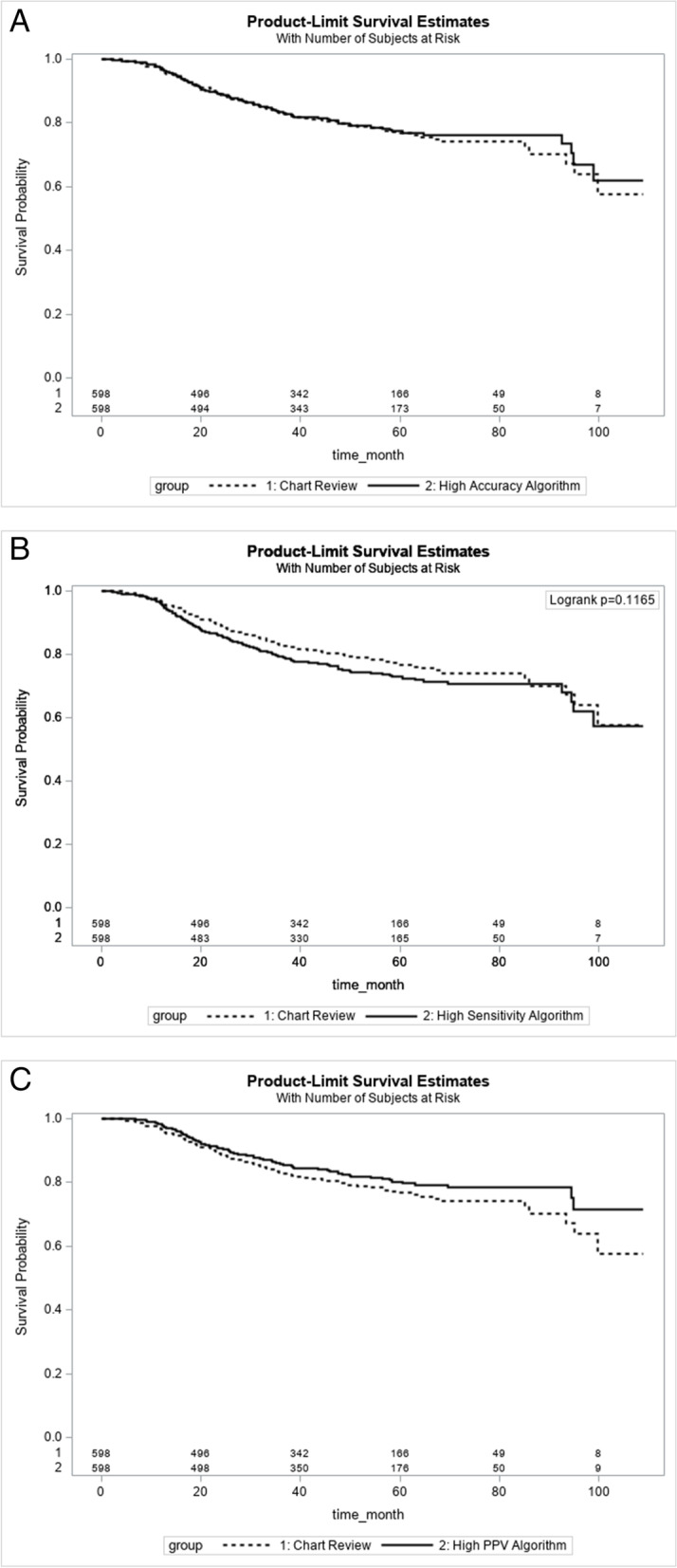
The comparison between estimated and chart review derived K-M curves for RFS. **A** shows the comparison of K-M curves between the high sensitivity algorithm estimated and chart review data with logrank *p*-value = 0.117; **B** shows the comparison of K-M curves between the high PPV algorithm estimated and chart review data with logrank *p*-value = 0.111; **C** shows the comparison of K-M curves between the high accuracy algorithm estimated and chart review data with logrank *p*-value = 0.729

There was also high agreement between the estimated and chart review generated K-M curves (Fig.1). There were no significant differences in 5-year RFS between the chart review (76.7, 95% CI: 74.7–78.8 months) and algorithm-estimated data with 72.4 (70.3–74.5) months for the high sensitivity algorithm, 79.8 (77.8–81.8) months for the high PPV algorithm, and 77.0 (75.0–79.0) months for the high accuracy algorithm. Beyond 5 years, slight divergence between algorithm-estimated and chart review K-M curves was observed, which was more apparent in the comparison between the high PPV algorithm and Chart-review curves. For the chart-review data, 477 (79.8%) patients were censored, of which there were 92 all-cause deaths (including 76 cancer-caused deaths). The number of censored patients was 482 (80.6%), 454 (75.9%), and 499 (83.4%), for the high-accuracy, high-sensitivity, and high-PPV algorithm, respectively.

The results of Cox regression of RFS (Table 3) showed high similarities between the estimated and chart review data in terms of the hazard ratio and the p-value of the independent variables. For the majority of the independent variables (e.g. age, year of diagnosis, HR status, HER2 status tumor grade, stage of tumor, histology, chemotherapy), the hazard ratio was consistent between chart review and algorithm-estimated data. Specifically, the hazard ratios with value of > 1.0 in the chart review data was still > 1.0 in the algorithm-estimated data, and the hazard ratios with value of < 1.0 in the chart review data remained < 1.0 in the algorithm-estimated data, except for ‘hormone therapy’ and ‘radiotherapy’ (both had a 95% CI crossing 1.0).

**Table 3:** The comparison of Cox regression of RFS between chart review and algorithm determined cohort

**Variable**		**Chart view** **HR (95% CI)**	***P*-value**	**High-accuracy** **HR (95% CI)**	***P*-value**	**High-PPV** **HR (95% CI)**	***P*-value**	**High-sensitivity** **HR (95% CI)**	***P*-value**
Age (year)	< = 35	Reference	0.124	Reference	0.123	Reference	0.125	Reference	0.162
	36–40	1.51 (0.89–2.57)		1.62 (0.92–2.84)		1.42 (0.78–2.57)		1.39 (0.85–2.27)	
	41–55	0.92 (0.51–1.66)		0.87 (0.47–1.6)		0.83 (0.44–1.6)		0.75 (0.44–1.29)	
	> = 56	0.66 (0.33–1.32)		0.7 (0.35–1.4)		0.53 (0.25–1.16)		0.72 (0.4–1.31)	
Tumor stage*	0-I	Reference	< 0.0001	Reference	< 0.0001	Reference	< 0.0001	Reference	< 0.0001
	II	1.83 (0.82–4.06)		2.92 (1.08–7.91)		1.84 (0.71–4.78)		2.3 (1.09–4.87)	
	III	7.19 (3.24–15.92)		11.83 (4.38–31.91)		8.62 (3.37–22.03)		8.19 (3.85–17.43)	
ER status	Negative	Reference	0.622	Reference	0.181	Reference	0.397	Reference	0.229
	Positive	0.87 (0.5–1.52)		0.69 (0.41–1.19)		0.77 (0.43–1.4)		0.74 (0.45–1.21)	
PR status	Negative	Reference	0.199	Reference	0.074	Reference	0.672	Reference	0.31
	Positive	0.71 (0.43–1.19)		0.63 (0.38–1.05)		0.89 (0.51–1.55)		0.79 (0.49–1.25)	
HER2 status*	Negative	Reference	0.003	Reference	0.003	Reference	0.007	Reference	0.016
	Positive	0.48 (0.3–0.77)		0.48 (0.3–0.77)		0.5 (0.3–0.83)		0.6 (0.4–0.91)	
Histology	Ductal	Reference	0.182	Reference	0.058	Reference	0.102	Reference	0.102
	Lobular	0.52 (0.2–1.36)		0.9 (0.37–2.2)		0.97 (0.39–2.43)		0.57 (0.24–1.38)	
	Other	1.51 (0.75–3.01)		2.17 (1.13–4.17)		2.16 (1.06–4.41)		1.67 (0.9–3.08)	
Tumor grade	1	Reference	0.379	Reference	0.052	Reference	0.065	Reference	0.101
	2	1.92 (0.75–4.93)		2.19 (0.76–6.3)		1.68 (0.58–4.88)		1.83 (0.77–4.38)	
	3	1.92 (0.75–4.93)		3.12 (1.0–8.86)		2.66 (0.93–7.63)		2.38 (0.99–5.66)	
Hormone therapy*	No	Reference	0.013	Reference	0.002	Reference	0.016	Reference	0.05
	Yes	0.74 (0.4–1.36)		1.22 (0.64–2.36)		0.84 (0.42–1.67)		0.99 (0.57–1.72)	
	Unknown	3.65 (1.35–9.81)		5.14 (2.06–12.83)		4.34 (1.47–12.79)		2.72 (1.16–6.35)	
Radiotherapy*	No	Reference	0.017	Reference	0.002	Reference	0.030	Reference	0.058
	Yes	1.01 (0.55–1.87)		1.03 (0.55–1.96)		0.88 (0.44–1.75)		0.97 (0.56–1.69)	
	Unknown	0.25 (0.1–0.66)		0.22 (0.09–0.52)		0.24 (0.09–0.7)		0.38 (0.17–0.85)	
Chemotherapy	No	Reference	0.158	Reference	0.216	Reference	0.258	Reference	0.001
	Yes	0.57 (0.27–1.24)		0.57 (0.24–1.38)		0.59 (0.24–1.47)		0.33 (0.17–0.63)^	
Year of diagnosis	2007–2009	Reference	0.784	Reference	0.228	Reference	0.394	Reference	0.188
	2010–2012	1.29 (0.63–2.63)		1.7 (0.82–3.52)		1.07 (0.48–2.39)		1.78 (0.96–3.31)	
	2013–2015	1.27 (0.56–2.88)		2.07 (0.9–4.75)		1.55 (0.63–3.79)		1.71 (0.83–3.54)	

*HR* hazard ratio, *CI* confidence interval, *PPV* positive predictive value, *ER* estrogen receptor, *PR* progesterone receptor, *HER2* Human Epidermal growth factor Receptor 2

* indicates that the variable was statistically significant (*p*-value < 0.05) based on chart review data

^ indicates that the variable was statistically significant (*p*-value < 0.05) based on high-sensitivity algorithm estimated data

In addition, most of the corresponding *p*-values of the hazard ratios of the variables were consistent between the chart review data and the algorithm-estimated data (Table 3). Except for the high-sensitivity algorithm-estimated ‘chemotherapy’, all the statistically significant (*P* < 0.05) hazard ratios derived from chart review data remained significant in algorithm-estimated data, and all statistically non-significant hazard ratios remained non-significant.

## Discussion

In the present study, we established new methods to identify the length of RFS of breast cancer patients using population-based real-world health data from a universal single-payer health system. Compared with the reference (chart review) data, the length of RFS determined by the developed algorithms achieved similar results, with the majority of the algorithm-estimated recurrence dates falling within 3 months of the real (i.e. chart review) recurrence dates. Moreover, the survival analyses created by both algorithm-estimated and chart review data showed high levels of correlation. This provides us with confidence to apply the developed algorithms in real RFS analyses. The developed methods have the potential to facilitate numerous down-stream research such as healthcare quality assessment, treatment efficacy comparisons, and decision-making support for patients with breast cancer. The method also provides a framework for constructing similar algorithms for identifying RFS of other cancers.

Previous studies [4–12] attempting to identify breast cancer recurrence and timing of recurrence vary by method and data used. The majority of these studies focused on identifying the recurrence status but not the timing of cancer recurrence. A few studies [5, 10–12] addressed the issue of determining the timing of recurrence by applying a prediction-model based methodology which incorporates a list of recurrence indicator variables to assign a probability of recurrence to each patient, then set a cutoff for the probability to classify recurrence vs. non-recurrence. Ritzwoller et al. [10] developed recurrence identification algorithms based on multivariable logistic regression models using data derived from several distinct but integrated health care delivery settings in the U.S., and reported 60–70% estimated dates of recurrence falling within ± 6 months of the true date of recurrence. This result is lower than in our study which found 80% of estimated dates falling within 6 months of chart review recurrence dates. Chubak et al. [5] developed a set of rule-based recurrence identification algorithms for breast cancer based on the Surveillance, Epidemiology and End Results (SEER) program cancer registry data and claims data, and reported a higher accuracy in terms of the date of recurrence estimation with 80% within ± 60 days of true recurrence date. One Danish study conducted by Rasmussen et al. developed rule-based algorithms based on the data from four Danish national registries. The authors reported better performed algorithms than the U.S. studies, but the superior performance that the author explained can be attributed to the inclusion of pathology codes and dates which is relatively specific to their data and not applicable in our population-based datasets.

Instead of developing new algorithms, we intended to validate the previously developed United States algorithms; however, a number of key differences in data impeded their application in our data. In addition to the United States specific coding systems including the Current Procedural Terminology (CPT) and Healthcare Common Procedure Coding System (HCPCS), one extremely contributable variable in the developed United States algorithms was the ‘second malignant diagnosis codes/records’ which was limited or not used in our data. In our data a ‘breast cancer diagnosis code’ (which is the same as the primary instance of cancer) was more commonly used instead of a ‘secondary malignant breast cancer code’ if the patient was previously diagnosed with breast cancer. Therefore, the inherent ability to identify cancer recurrence directly is not possible in most Canadian population-based datasets. Therefore, we decided to develop new algorithms to address this issue and others that have hindered analysis with these datasets previously.

Generally, the K-M curves generated using chart review and algorithm estimated data were very similar. While before 5-year follow-up, the high-accuracy algorithm generated K-M curve was highly consistent with that of chart-review, a slightly higher RFS rate was observed in the high-accuracy algorithm curve after 5-years follow-up. This may be due to that the high-accuracy algorithm estimated a delayed recurrence date for some patients, or missed some patients who had a recurrence after 5-year follow-up. This also explains the higher RFS in the high-PPV algorithm K-M curve than that of chart-review curve, given that high-PPV algorithm tended to miss recurrence to ensure the high PPV. Conversely, to ensure high sensitivity, the high-sensitivity algorithm tended to identify more false recurrent cases at the early years of follow-up, thus, a lower RFS rate was observed as compared to chart review K-M curve.

In addition to directly comparing the estimated RFS and chart review RFS, we also conducted comparisons between them by assessing the similarities in survival analyses between the estimated RFS and chart review RFS. Because the main application of the developed algorithm is enabling the RFS analysis, our comparison results demonstrated that the high accuracy algorithm and high PPV algorithm-estimated RFS data were reliable data for RFS analysis given that all the hazard ratios of independent variables were consistent with that of the chart review data. Worthy to note, only one variable’s hazard ratio (chemotherapy) based on the high sensitivity algorithm was not consistent with the chart review. The potential reason for this may be due to the fact that the majority (91.5%) of the patients had chemotherapy and only 10 patients had no chemotherapy among the patients with recurrence. Thus, a subtle misclassification of the algorithm can lead to a disproportional change between the recurrent and non-recurrent groups in terms of the number of patients undergoing chemotherapy, and then produced a high impact on the hazard ratio of chemotherapy. In the utility of RFS analysis, we recommend the high-accuracy algorithm which demonstrated the most similar results with that of the chart review data.

There were several limitations in our study which need to be considered. First, we used data from two previously chart reviewed cohorts who were deemed to have a high risk of breast cancer recurrence. Since the proposed algorithms were not validated in a random sample of our overall breast cancer population, we cannot guarantee that the proposed algorithm performances will be similar for other breast cancer cohorts. However, the pattern of medical encounter of the recurrent breast cancer patients in our cohort is generalizable to other breast cancer patient cohorts, thus the developed algorithms should be applicable to other breast cancer cohorts. Second, the proposed algorithms were not designed to differentiate between second primary breast cancer and breast cancer recurrence. Therefore, some of the recurrences identified by the proposed algorithm may have been second primary breast cancers. However, many cancer outcome studies consider both second primary breast cancer and breast cancer recurrence as the same event. Third, the proposed algorithm does not differentiate types of recurrence (e.g., non-invasive, local–regional, metastatic). Chart review would still be required if such information is needed; however, the algorithms will have significantly narrowed the task. Finally, the algorithms were only internally validated using our cohort. The performance of the algorithms is unclear when applied to external data. Thus, external validation using data from other provinces or nations with a universal health system is needed.

## Conclusion

By using widely available real-world health data, the proposed algorithm attained similar results to that of chart review in identifying the timing of recurrence among breast cancer patients in a universal health system. Furthermore, the algorithm estimated data generated similar results of RFS analysis with the chart review. This implies that the developed algorithms have the potential to replace chart review in real population-based RFS analyses. Additional work on external validation is necessary in the future.

## Previous presentations

Some preliminary results of the work have been presented at the 2019 Canadian Association of Health Services and Policy Research Annual Conference, Halifax, Canada; and the abstract was available online: https://cahspr.ca/wp-content/uploads/2020/11/Book-of-Abstracts-CAHSPR-2019.pdf.

## Supplementary Information

**Additional file 1: Table 1.** The codes used for defining the study variables

**Additional file 2: Fig. 1.** The algorithm with high sensitivity for identifying recurrence of breast cancer

**Additional file 3: Fig. 2.**The algorithm with high positive predictive value for identifying recurrence of breast cancer

**Additional file 4: Fig. 3.** The algorithm with high overall accuracy for identifying recurrence of breast cancer

## Data Availability

The datasets generated during and analyzed during the current study are available in this article and in the supplemental materials.

## Abbreviations

ACR: Alberta Cancer Registry
AHS: Alberta Health Services
BCS: Breast-conserving Surgery
CART: Classification and Regression Tree
CCCR: Calgary Centre for Clinical Research
CPT: Current Procedural Terminology
EMR: Electronic Medical Record
HER2: Human Epidermal Growth Factor Receptor-2
HR: Hormone Receptor
HCPCS: Healthcare Common Procedure Coding System
IQR: Interquartile Ratio
K-M: Kaplan-Meyer
PPV: Positive Predictive Value
RFS: Recurrence-free Survival
SEER: Surveillance, Epidemiology and End Results
