# Supplementary material for: New method for determining breast cancer recurrence-free survival using routinely collected real-world health data
Source: BMC Cancer. 2022 Mar 16;22:281. doi: 10.1186/s12885-022-09333-6 (PMC8925135; doi:10.1186/s12885-022-09333-6)
Supplement: Supplementary file 1 — Additional file 1: Table 1. The codes used for defining the study variables [file 12885_2022_9333_MOESM1_ESM.pdf]

Table 1: Codes used for defining the study variables

Study variable	Data source	Codes/specification
Breast surgery/procedure		
Mastectomy	Hospital discharge abstract, Ambulatory care reporting system, and physician billing claims data	CCI: 1YM89^^, 1YM90^^, 1YM91^^,1YM92^^<br>Physician billing codes: 97.12A, 97.12B, 97.21A, 97.22A
Breast conservative surgery		CCI: 1YM87^^, 1YM88^^<br>Alberta physician billing codes: 97.27A, 97.27B
Breast biopsy		CCI: 2JZ71^^, 2YM71^^, 2MD71^^, 2YR71^^, 2YS71^^<br>Physician billing codes: 97.81, 97.82A, 97.89A, 97.11A, 97.11B
Mammography		CCI: 3YM10^^<br>Physician billing codes: X26
Chemotherapy	Cancer center electronic medical records	Using the date of each administration of chemotherapy
Radiation therapy	Cancer center electronic medical records	Using the date of each radiation therapy
Referral to oncologist	Cancer center electronic medical records	Using the date of each referral to oncologist
Type of medical visit		
Cancer center visit	Cancer center electronic medical records	Based on the service facility we identified the cancer center visit
Specialty visits	Physician billing claims data	Based on the provider classification we identified the type of specialty including the oncologists and general surgeons
Primary cancer site (breast cancers)	Alberta cancer registry data	ICD-9: 174.^ and 233.^<br>ICD-10: C50.^ and D05.^

Death caused by cancer	Vital statistics data	ICD-9: 140.^ - 208.^ and 233^<br>ICD-10: C00.^ - C97.^ and D05.^

CCI: Canadian Classification of Health Intervention; ICD-9: International Classification of Disease – ninth edition; ICD-10: International Classification of Disease – tenth edition.
Note: Physician billing codes are derived from the Alberta Schedule of Medical Benefits (SOMB).
