# Supplementary material for: New method for determining breast cancer recurrence-free survival using routinely collected real-world health data
Source: BMC Cancer. 2022 Mar 16;22:281. doi: 10.1186/s12885-022-09333-6 (PMC8925135; doi:10.1186/s12885-022-09333-6)
Supplement: Supplementary file 2 — Additional file 2: Fig. 1. The algorithm with high sensitivity for identifying recurrence of breast cancer [file 12885_2022_9333_MOESM2_ESM.pdf]

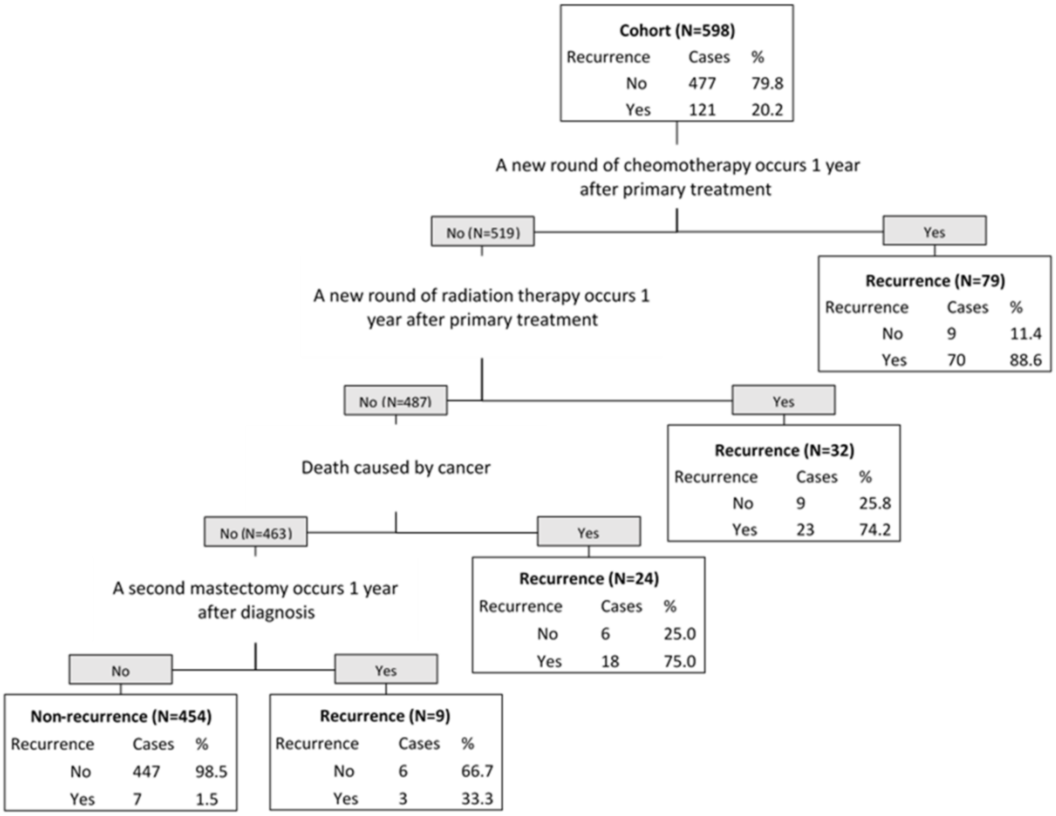

Figure 1: The algorithm with high sensitivity for identifying recurrence of breast cancer. “Yes” means the criteria was met; “No” means the criteria was not met.
