# Supplementary material for: New method for determining breast cancer recurrence-free survival using routinely collected real-world health data
Source: BMC Cancer. 2022 Mar 16;22:281. doi: 10.1186/s12885-022-09333-6 (PMC8925135; doi:10.1186/s12885-022-09333-6)
Supplement: Supplementary file 3 — Additional file 3: Fig. 2.The algorithm with high positive predictive value for identifying recurrence of breast cancer [file 12885_2022_9333_MOESM3_ESM.pdf]

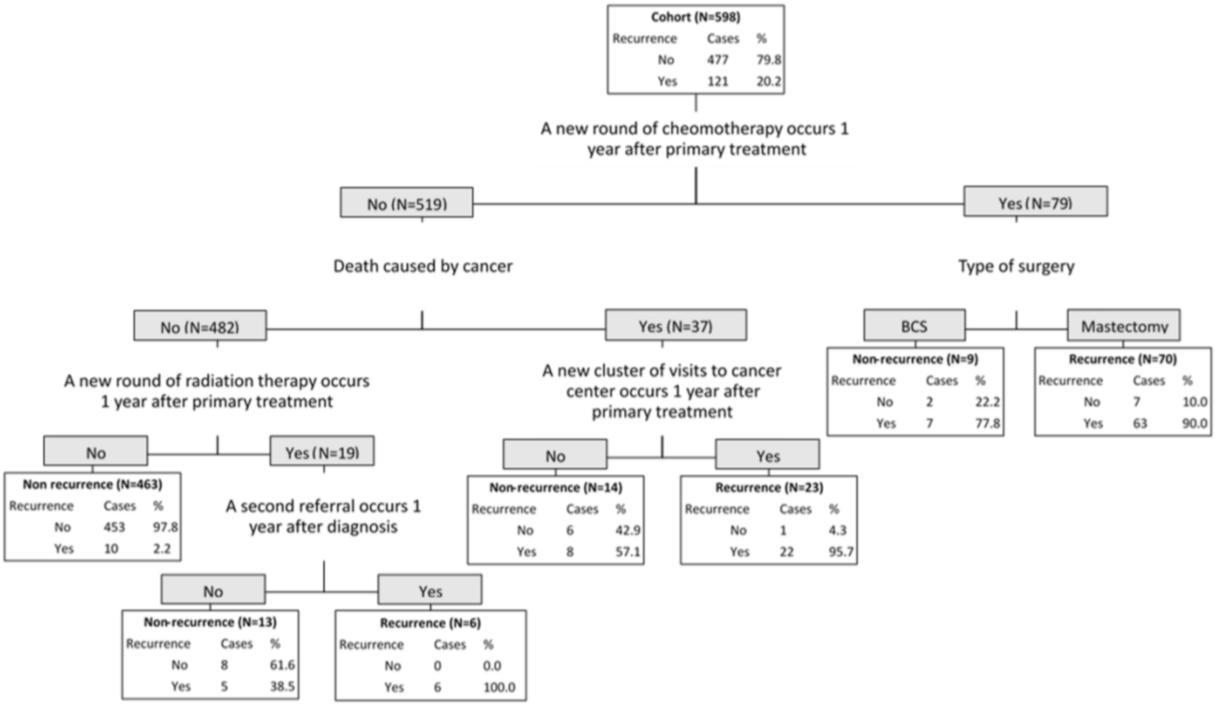

Figure 2: The algorithm with high positive predictive value for identifying recurrence of breast cancer.
"Yes" means the criteria was met; "No" means the criteria was not met.
